# Comparing the Quality of Ambulatory Surgical Care for Skin Cancer in a Veterans Affairs Clinic and a Fee-For-Service Practice Using Clinical and Patient-Reported Measures

**DOI:** 10.1371/journal.pone.0171253

**Published:** 2017-01-31

**Authors:** Matthew P. Dizon, Eleni Linos, Sarah T. Arron, Nancy K. Hills, Mary-Margaret Chren

**Affiliations:** 1 Program for Clinical Research, Department of Dermatology, University of California San Francisco, San Francisco, California, United States of America; 2 San Francisco Veterans Affairs Medical Center, San Francisco, California, United States of America; 3 Department of Biostatistics and Epidemiology, University of California San Francisco, San Francisco, California, United States of America; Uppsala University, SWEDEN

## Abstract

The Institute of Medicine has identified serious deficiencies in the measurement of cancer care quality, including the effects on quality of life and patient experience. Moreover, comparisons of quality in Veterans Affairs Medical Centers (VA) and other sites are timely now that many Veterans can choose where to seek care. To compare quality of ambulatory surgical care for keratinocyte carcinoma (KC) between a VA and fee-for-service (FFS) practice, we used unique clinical and patient-reported data from a comparative effectiveness study. Patients were enrolled in 1999–2000 and followed for a median of 7.2 years. The practices differed in a few process measures (e.g., median time between biopsy and treatment was 7.5 days longer at VA) but there were no substantial or consistent differences in clinical outcomes or a broad range of patient-reported outcomes. For example, 5-year tumor recurrence rates were equally low (3.6% [2.3–5.5] at VA and 3.4% [2.3–5.1] at FFS), and similar proportions of patients reported overall satisfaction at one year (78% at VA and 80% at FFS, P = 0.69). These results suggest that the quality of care for KC can be compared comprehensively in different health care systems, and suggest that quality of care for KC was similar at a VA and FFS setting.

## Introduction

In a landmark report, the Institute of Medicine (IOM) defined quality of care as safe, effective, patient-centered, timely, efficient, and equitable [1]. Within the field of cancer care specifically, the IOM more recently termed the measurement of quality a “crisis” with serious deficiencies in evidence on the effect of care on quality of life (QOL) and overall patient experience [2]. The linkage of population-based registries and Medicare survey databases will allow for more comprehensive comparisons of quality for many types of cancer; patients with the most common malignancies—basal cell carcinoma and cutaneous squamous cell carcinoma (collectively termed keratinocyte carcinoma, KC)—are routinely excluded from these registries, however, leading to a major gap in dermatologic quality-of-care research [3, 4].

Our goal was to use unique clinical and patient-reported data from a comparative effectiveness study of keratinocyte carcinoma to compare quality of ambulatory surgical care for cancer between a government-operated managed-care facility (VA) and a fee-for-service (FFS) practice.

## Methods

### Overview

With the exception of high-risk squamous cell carcinoma in immunocompromised patients, keratinocyte carcinomas (KCs) only rarely metastasize and are typically treated surgically; the most common treatments are tumor destruction with electrodesiccation and curettage, excision, and Mohs micrographic surgery (intraoperative histologically guided tumor removal). Nonsurgical treatments, such as topical chemotherapy, topical immunomodulators, and photodynamic therapy, were rarely used in this study. Conventionally, the primary goal of treatment has been complete tumor eradication to prevent local recurrence, although cosmetic outcomes are important since these tumors often occur on the face and other visible areas of the body. For typical KCs, most therapies are curative, but there is variation in the use of therapies in different practice settings [5,6].

**Fig 1:**
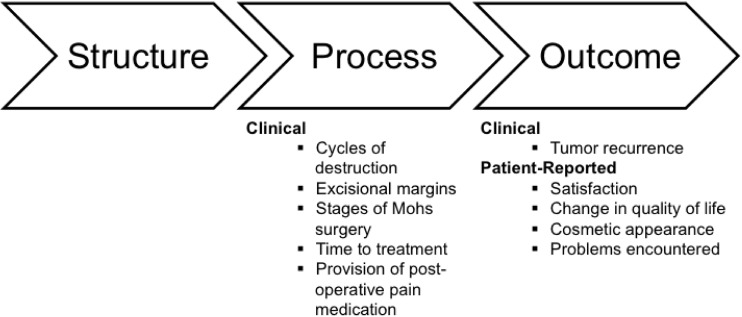
Donabedian Model for quality of care adapted for care of KC.

Due to the complexity of cancer care, quality cannot be summarized adequately by a single composite measure [7]. We adopted the Donabedian Model (Fig 1) [8,9] and examined quality of care in terms of processes of care, clinical outcomes, and patient-reported outcomes.

### Design, Setting, Patients, Baseline Data

In this study, we utilized data from a prospective cohort study of all patients with KC diagnosed in 1999–2000 and treated in a fee-for-service dermatology practice or a VA dermatology practice. Both practices were staffed by providers from the same academic institution, however, the majority of providers practiced in only one setting. The study was approved by the University of California, San Francisco Human Research Protection Program Laurel Heights Institutional Review Board Committee, and when required, patients provided written informed consent.

Details of the study have been described previously [5,10–12]. The parent study was powered to detect a difference of 4% in 5-year tumor recurrence between treatments. Eligible patients were all patients with KC, defined histopathologically as basal cell carcinoma or squamous cell carcinoma of the skin. The sample was restricted to tumors treated with any of the three most common therapies (electrodesiccation and curettage, excision, and Mohs surgery). Patients with basal cell nevus syndrome were excluded (S1 and S2 Figs).

### Data Collection

The primary source of clinical data was the medical record. In the parent study, trained nurse practitioners who were blinded to study goals and as much as possible to treatment type reviewed all records using structured data forms at a median of 9.0 years after treatment. A dermatologist blinded to treatment type also examined patients who consented at a median of 8.6 years after treatment.

The primary sources of data on patients’ reports were surveys, administered before therapy and periodically afterward.

### Measures

#### Process measures

Clinical process measures were derived through review of patient records. For tumors treated by destruction, the number of cycles of electrodesiccation and curettage was recorded. For tumors treated by excision, the sizes of surgical margins were measured in millimeters. For tumors treated by Mohs surgery, the number of removed tissue layers (called stages) was recorded. The time to treatment was defined as the number of days between initial biopsy and treatment. Post-operative pain medication data was recorded as acetaminophen/hydrocodone, acetaminophen with codeine, other, or none noted. Each patient’s total number of follow-up visits to the dermatology practices was counted and used to calculate the average number of annual dermatology visits per patient during the follow-up period.

#### Primary clinical outcome measure

A tumor was defined as recurrent if the tumor type (basal cell carcinoma or squamous cell carcinoma) was identical to the primary tumor, the body location was identical or very close to that of the primary tumor, and the lesion was described by the clinician as recurrent or previously treated. Examination of the patient record included review of lesion maps and photographic documentation, when available. Although photographic documentation was used often, its use in individual cases was not recorded. For time to recurrence, date of initial treatment was used as the time of origin, and the date of recurrence was defined as the date of biopsy of the recurrent lesion. Secondary recurrences were rare so only the first recurrence was included in the analysis. Data were right-censored at the last date of care. For each tumor, follow-up ended at the last date when the patient received care. A patient was lost to follow up if there was no record of care after treatment.

#### Patient-reported outcome measures

Patient satisfaction at three months after treatment was measured with an adapted version of the validated Patient Satisfaction Questionnaire-18 (PSQ-18) [13,14]. The PSQ-18 measures general satisfaction as well as satisfaction with six domains of care. Using a 5-item response scale, patients responded about how strongly they agreed or disagreed with statements about the technical quality, interpersonal manner, communication, financial aspects, time spent with the clinician, and accessibility, as well as their general satisfaction. Patient satisfaction at one year after treatment was measured using two global questions about how strongly patients agreed or disagreed with the statements, “I am completely satisfied with the treatment of my skin problem," and “I am completely satisfied with the follow-up care for my skin problem.”

Skin-related QOL was measured by a validated tool, the Skindex-16 [15]. Cosmetic outcome was measured with a single global question, “How would you describe the cosmetic outcomes (appearance) of your skin treatment?” using a 5-item response scale ranging from “Poor” to “Excellent.” Problems encountered during treatment were measured with a single global question, “In your opinion, have there been any complications of the treatment of your skin problem?” Responses were recorded as “Yes” or “No.”

Global items measuring cosmetic outcomes, problems encountered, satisfaction with care overall, and satisfaction with follow-up care were composed specifically for this study and have not been externally validated. All of these study-specific global measures utilized 5-item response scales. For example, the measure for cosmetic outcome included 5 options ranging from “Poor” to “Excellent.”

#### Additional variables

Baseline mental health status was measured with an adapted version of the Medical Outcomes Study Short Form-12 instrument’s Mental Component Score [16]. A single item from the Short-Form 12 was used to measure self-reported general health status. Age, gender, previous history of KC, and number of KC lesions at presentation were obtained from the medical record. Education and race were obtained from pretreatment patient surveys. Tumors were classified according to the presence of risk factors for recurrence [17,18], including location, size, definition of borders, whether a tumor was primary versus recurrent, immunosuppression, prior radiotherapy, and histological risk factors.

### Analytic Strategy

Based on previous studies and our clinical experience, we hypothesized that 5-year tumor recurrence rates, frequency of reported problems, cosmetic outcomes, and change in QOL would be similar in the two practice settings. We also hypothesized that patients in the FFS practice would report higher satisfaction with technical skills, personal manner, and communication by providers, but lower satisfaction with the cost of their care than patients treated in the VA practice [19–22].

Statistical analyses were performed using Stata version 14.1. We compared patient and tumor characteristics between the two sites, using Chi^2^ tests for binary categorical characteristics and Wilcoxon-rank sum tests for ordinal characteristics. The same tests were used to compare clinical process measures.

The primary clinical outcome of tumor recurrence was displayed using Kaplan-Meier plots for the entire sample and in treatment subgroups. We determined unadjusted 5-year recurrence rates by practice setting and compared survivor functions using a log-rank test. We performed a Cox proportional hazard model to calculate a hazard ratio after adjustment for characteristics likely related to recurrence. The proportional-hazards assumption was tested on the basis of Schoenfeld residuals after fitting the model. Due to the limited number of recurrences, individual risk factors defined by the National Comprehensive Cancer Network guidelines [17,18] were combined into a binary independent variable, which categorized tumors as high or low risk for recurrence. In addition to risk for recurrence, we included treatment type, frequency of dermatology follow-up, and having multiple KC lesions at presentation as additional variables in the model as they were found to be predictive of recurrence in previous studies. Unadjusted Cox proportional hazard models were also performed with a single predictor of practice setting; results were similar and are not reported. The models were fit with clustering on provider to account for intra-provider correlations; results were also similar and are not reported.

Patient-reported measures were dichotomized with cut-off values that we deemed most reasonable based on the wording of response options. Satisfaction scores were dichotomized as “agree/strongly agree” vs. all others. Change in skin-related QOL was measured as the difference between Skindex-16 domain scores at pre-treatment and 1-year after treatment; improvement was defined as a reduction in score of 10 points or greater. Cosmetic outcome responses were dichotomized as “good/very good/excellent” vs. “poor/fair.” Problems encountered during or up to 1-year after treatment had the binary response of “Yes” or “No.”

The proportions of patients reporting each response were compared between sites in unadjusted analyses using Chi^2^ tests. We then fit logistic regression models in which the major predictor variable was practice setting, adjusting for type of treatment and patient-mix variables that are used in standard consumer assessments such as the Consumer Assessment of Healthcare Providers and Systems (CAHPS) Hospital Survey [23], including age, gender, race, education, and general and mental health status. Models for change in skin-related QOL also included baseline Skindex-16 domain scores. Measures with missing data were subject to listwise deletion. Unadjusted logistic regression models for the patient reports were also performed with a single predictor of practice setting; results were similar and are not reported. Models were also fit using generalized estimating equations to account for within-provider correlations; results were also similar and are not reported.

## Results

### Study Characteristics

The study enrolled 1353 eligible patients with KCs who were treated with destruction, excision, or Mohs surgery. VA patients were older, more likely to be male, had lower levels of education, worse self-reported general health, and were more likely to have had a previous history of KC. Tumors treated at the VA were more likely to be treated with excision compared to tumors treated at the FFS, as we have reported previously [12] (Table 1). Although Mohs surgery was available at both sites, one weekly clinical session was devoted to Mohs surgery at the VA while the FFS practice had two to three sessions per week, indicating that this service might have been less readily available.

**Table 1:** Patient and Tumor Characteristics.

			VA (n = 562)		FFS (n = 791)	
			n	(%)	n	(%)
Patient characteristics						
	Age, mean (sd)		71.2	11	62.9	16
	Male sex		541	96	450	57
	Education completed					
		Elementary school	36	10	8	2
		High school	177	49	90	21
		College	78	22	165	38
		Graduate/Professional	72	20	170	39
	White race		348	94	404	94
	Self-reported general health					
		Excellent	23	6	75	17
		Very good	76	20	135	31
		Good	157	41	140	32
		Fair	98	26	69	16
		Poor	31	8	18	4
	Mental component summary					
		Mean (sd)	48 (11.8)		48.7 (10.5)	
		Median (IQR)	50.5 (39.2–57.8)		51.6 (41.8–56.9)	
	Previous history of KC		328	58	408	52
	Number of KCs at presentation					
		One	464	83	638	81
		Multiple	98	17	153	19
Tumor characteristics			(n = 706)		(n = 1024)	
	Histological category					
		SCC	191	27	209	20
		High risk SCC type (adenoid, pseudoglandular, acantholytic, metatypical, desmoplastic, adenosquamous)	13	7	13	6
		BCC	515	73	815	80
		High risk BCC type (micronodular, infiltrative, morpheaform, desmoplastic, sclerosing, basosquamous/metatypical)	110	21	237	29
	Recurrence risk (NCCN 2016)					
		High	448	63	616	60
		Low	258	37	408	40
	Treatment type					
		Destruction	139	20	475	26
		Excision	370	52	284	28
		Mohs surgery	197	28	265	46

Missing (VA/FFS): Education (199/358), race (193/363), self-reported general health (177/354), MCS-12 (217/376), dermatology follow-up (7/75). Abbreviations: Veterans Affairs (VA), fee-for-service (FFS), keratinocyte carcinoma (KC), basal cell carcinoma (BCC), cutaneous squamous cell carcinoma (SCC), standard deviation (sd), interquartile range (IQR), National Comprehensive Cancer Network (NCCN).

### Process Measures

Although frequency of use of treatments differed in the two settings [5], treatments were performed similarly in both settings. For example, there was no significant difference in the median size of surgical margins for tumors treated with excision (3 mm (IQR 3–4) at both sites, P = 0.84) or median number of stages for tumors treated with Mohs surgery (2 (IQR 1–2) at both sites, P = 0.18). The time between biopsy and treatment was significantly longer at the VA compared to FFS (median 46 days versus 38.5, respectively, P<0.001). Patients in the FFS setting were more likely to use post-operative pain medication (44% versus 17%, P<0.001) and VA patients had more frequent follow-up with dermatology (53% of patients at the VA had greater than 2 annual visits compared to 35% at the FFS, P<0.001) (Table 2).

**Table 2:** Processes of Care.

	VA		FFS		
	Median	(IQR)	Median	(IQR)	P-value
Electrodesiccation and curettage, no. of cycles	3	(3–3)	3	(3–3)	0.001
Excision, size of margins (mm)	3	(3–4)	3	(3–4)	0.84
Mohs surgery, no. of stages	2	(1–2)	2	(1–2)	0.18
Days between biopsy and treatment	46	(27–75)	38.5	(21–57)	<0.001
Provision of post-operative pain medication, %	115	17%	447	44%	<0.001*
Annual dermatology visits, % > 2	699	53%	928	35%	<0.001*

Missing (VA/FFS): ED&C cycles (55/22), excision margins (198/129), Mohs surgery stages (5/4), pain medication (11/15), annual dermatology visits (7/96).

*P-value calculated with Chi^2^ test, all other p-values were calculated with Wilcoxon rank-sum test. Abbreviations: Veterans Affairs (VA), fee-for-service (FFS), interquartile range (IQR), electrodesiccation and curettage (ED&C).

### Clinical Outcomes

**Fig 2:**
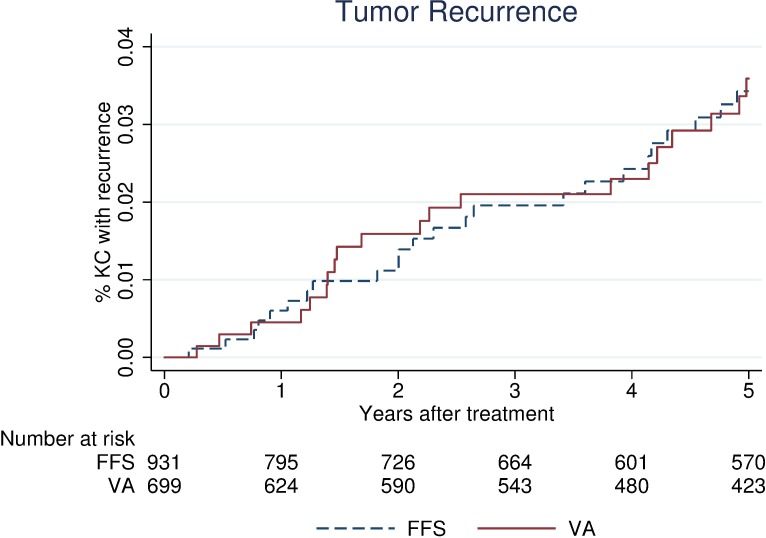
Clinical Outcome Measure, Kaplan-Meier failure curve for tumor recurrence. Abbreviations: Veterans Affairs (VA), fee-for-service (FFS), keratinocyte carcinoma (KC).

Follow-up about tumor recurrence was available for 94% of tumors. Five years after treatment, 44 tumors recurred (Fig 2). Unadjusted 5-year recurrence rates did not differ significantly between practice settings: 3.6% [2.3–5.5] at the VA and 3.4% [2.3–5.1] (P = 0.88) at the FFS. This finding was consistent in a Cox proportional hazard model, which included treatment type, risk for recurrence [17,18], multiple KCs at presentation, and >2 annual dermatology visits during the follow-up period: hazard ratio of 0.79 [0.42–1.49] (P = 0.46) at the VA compared to FFS. Kaplan-Meier curves for tumor recurrence by treatment type were not significantly different (S3 Fig).

### Patient-Reported Outcomes

Three months after treatment, the majority of patients in both settings reported satisfaction with most domains of care (Table 3). The proportion of patients reporting satisfaction with technical quality (i.e., whether patients felt confident in their providers’ abilities, that they were examined carefully, and necessary resources and equipment were available in the office) was lower in both settings compared to other domains of satisfaction: 52% (VA) and 48% (FFS) (P = 0.43). The two settings significantly differed only in the domain of accessibility (i.e., availability of appointments, specialists, and emergency treatment): VA patients were more likely to report satisfaction with accessibility (59% (VA) versus 43% (FFS), P <0.001).

**Table 3:** Patient-Reported Outcome Measures.

	Unadjusted Proportions					Multivariable Adjusted Odds***		
	VA		FFS			VA:FFS		
Satisfaction, Satisfied—Scores >4	n	%	n	%	P-value*	Odds Ratio	95% CI	P-Value**
Three months								
General Satisfaction	209	73	216	73	0.92	1.18	0.70–1.98	0.54
Technical Quality	147	52	143	48	0.43	0.81	0.51–1.28	0.36
Interpersonal Manner	233	82	254	86	0.18	0.73	0.38–1.41	0.35
Communication	222	78	247	83	0.09	1	0.55–1.82	0.99
Financial Aspects	230	82	224	76	0.09	1.59	0.91–2.77	0.11
Time Spent with MD	206	73	203	69	0.32	1.49	0.89–2.49	0.13
Accessibility	169	59	127	43	<0.001	1.98	1.24–3.17	<0.001
One-year								
Global Satisfaction	211	78	237	80	0.69	0.81	0.44–1.49	0.5
Satisfaction with follow-up	210	77	231	79	0.76	0.75	0.42–1.35	0.34
Quality of life, 10-point improvement or not****								
One-year								
Emotions domain	131	54	125	52	0.58	1.14	0.63–2.06	0.66
Symptoms domain	91	38	72	30	0.07	0.99	0.50–1.94	0.97
Functioning Domain	58	24	45	19	0.18	1.12	0.54–2.32	0.77
Other								
One-year								
Favorable cosmetic outcome	224	84	244	83	0.84	1.15	0.59–2.22	0.68
Encountered problems with care	47	18	41	14	0.18	1.08	0.57–2.07	0.81

Missing values (VA/FFS): Satisfaction 3 months (100-103/141-144), Global satisfaction 1 year (116/140), Satisfaction with follow-up (114/143), Change in QOL (142-147/196-200), Cosmetic appearance (118/144), Encountered problems (129/146).

*P-value calculated using the Chi^2^ test.

**P-value calculated using the Wald Chi^2^ test.

***Multivariable logistic regression models adjusted for treatment type (3 strata, categorical), age (7 strata, categorical), education (4 strata, categorical), self-reported general health status (5 strata, categorical), sex, race (white or non-white), and Mental Component Summary scores (7 strata, categorical).

****Models for Quality of Life included baseline Skindex-16 domain scores (4 strata, categorical). Abbreviations: Veterans Affairs (VA), fee-for-service (FFS).

One year after treatment, the majority of patients reported overall satisfaction with care in both settings: 78% (VA) and 80% (FFS) (P = 0.69). Similarly, satisfaction with follow-up care reported by the majority of patients in both settings: 78% (VA) and 79% (FFS) (P = 0.76). The proportions of patients reporting improvement in skin-related QOL, cosmetic appearance, and problems encountered during care at one year after treatment were also not significantly different in the two settings.

These findings were consistent after adjustment for type of treatment and standard patient-mix characteristics including age, education, self-reported general and mental health, race, and gender.

## Discussion

Comparisons of quality of care in different practice settings may inform both health policy decisions and purchasers’ choices among plans [24]. We were able to compare ambulatory surgical care for KC in different health care settings using both clinical and a broad array of patient-reported measures. Care for KC in a VA and a FFS practice setting differed in few process measures, but we were unable to detect any substantial or consistent differences in clinical or patient-reported outcomes. For example, VA patients waited longer between biopsy and treatment but were more satisfied with the accessibility of care. Five-year recurrence rates were equally low in both settings and the majority of patients were satisfied with most domains of care, experienced improvement in skin-related quality of life, rated their cosmetic outcomes favorably, and encountered no problems with care.

Most comparative studies of quality of care have focused on either clinical measures of quality or patients’ reports of their experiences of care, but few studies have examined both dimensions simultaneously [25]. For nonfatal conditions like KC, patient-reported measures of performance are especially important [26]. Similar to this study, systematic reviews of comparisons of quality of care using clinical measures also have not detected significant differences in outcomes in VA and non-VA practices despite significant differences in adherence to accepted processes of care [21,22]. Patient-reported measures of quality have also been found to vary across practice settings [19,20] with overall ratings of care favoring FFS practices. These studies, however, were not specific to ambulatory surgical care for skin cancer.

### Potential Limitations

The study utilizes data about KC care for in 1999–2000 with follow-up through 2011; we think it unlikely, however, that the quality of care for KC has changed significantly during that time period. We made the comparisons now because we realized they provide a unique opportunity to fill a gap in dermatologic quality-of-care research. The recent availability of linked datasets that combine Surveillance, Epidemiology, and End Results Program (SEER) clinical cancer registry data with patients’ reports from the Medicare Health Outcomes Survey (MHOS) or Consumer Assessment of Healthcare Providers and Systems (CAHPS) surveys excludes patients with KC, so the quality of care for KC in different practice settings remains a significant gap that contemporary linked datasets cannot fill. Moreover, this comparison is timely now that the Veterans Access, Choice and Accountability Act of 2014 [27] permits some Veterans to elect to receive VA-funded care in eligible non-VA settings.

Although the cohort is large, consecutive, and typical of KC patients nationwide [28–30], the sample was assembled in a single city and treated by providers from the same academic institution, so the results may not be generalizable more broadly. For example, departmental policies and hiring practices may have minimized provider-level differences in quality that may exist between other VA and non-VA settings. Our study, however, would still be able to detect differences in quality mediated by systems-level factors.

We did not study the use of non-surgical topical therapies, which are now approved for treatment of some superficial tumors (limited to the epidermis only). In addition, because practice setting was not assigned randomly, unmeasured characteristics may have confounded the relationship between practice setting, tumor recurrence, and patients’ reports. However, we did collect patient-mix characteristics typically used in current quality assessments as well as established risk factors for tumor recurrence [17,18].

Although the PSQ-18 measures several domains of care, it does not differentiate care between nurses, doctors, and non-provider hospital staff. The PSQ-18 also does not measure satisfaction with pain management or cleanliness and quietness of the hospital environment, which are important aspects of quality measured in the modern HCAHPS survey.

### Conclusion

Quality of ambulatory surgical care for KC, assessed by both clinical and patient-reported measures, was similar in a government-operated managed care practice and a fee-for-service practice. This finding was consistent even after adjustment for established risk factors for tumor recurrence and patient-mix characteristics typically measured in standard consumer assessments. To our knowledge, this study is the first to compare the quality of ambulatory surgical care for skin cancer across practice settings using comprehensive measures, and fills a gap in knowledge about care for the most common cancer.

## Supporting Information

S1 Fig Patients’ reports sample derivation. (EPS)

S2 Fig Tumor recurrence sample derivation. (EPS)

S3 Fig Five-year tumor recurrence. (a) Electrodesiccation and curettage, (b) Excision, and (c) Mohs surgery.
(TIF)

## Abbreviations

BCC: basal cell carcinoma
SCC: cutaneous squamous cell carcinoma
CAHPS: Consumer Assessment of Healthcare Providers and Systems
ED&C: electrodesiccation and curettage
FFS: fee-for-service
KC: keratinocyte carcinoma
NCCN: National Comprehensive Cancer Network
PSQ-18: Patient Satisfaction Questionnaire-18
QOL: quality of life
VA: Veterans Affairs
