# Supplementary figures and images for: Comparing the Quality of Ambulatory Surgical Care for Skin Cancer in a Veterans Affairs Clinic and a Fee-For-Service Practice Using Clinical and Patient-Reported Measures
Source: PLoS One. 2017 Jan 31;12(1):e0171253. doi: 10.1371/journal.pone.0171253 (PMC5283736; doi:10.1371/journal.pone.0171253)

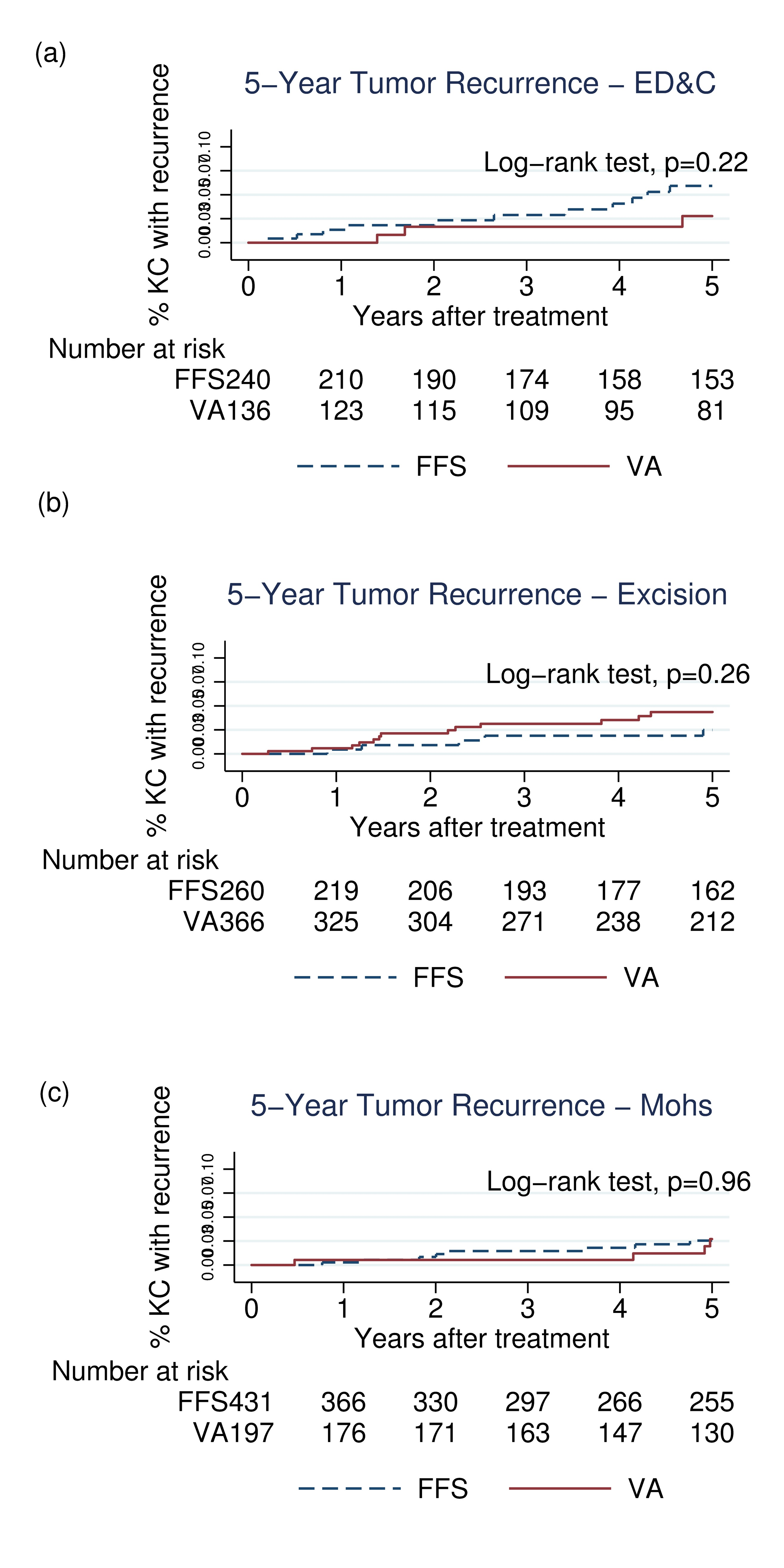

Supplement: S3 Fig — (a) Electrodesiccation and curettage, (b) Excision, and (c) Mohs surgery. (TIF) [file pone.0171253.s003.tif]
